# Magnetic resonance fingerprinting: from evolution to clinical applications

**DOI:** 10.1002/jmrs.413

**Published:** 2020-06-28

**Authors:** Jean J. L. Hsieh, Imants Svalbe

**Affiliations:** ^1^ Department of Diagnostic Radiology Tan Tock Seng Hospital Singapore Singapore; ^2^ Department of Medical Imaging and Radiation Sciences Monash University Clayton Victoria Australia; ^3^ School of Physics and Astronomy Monash University Clayton Victoria Australia

**Keywords:** Biomarkers, magnetic resonance imaging, MR fingerprinting, quantitative MRI, reproducibility of results

## Abstract

In 2013, Magnetic Resonance Fingerprinting (MRF) emerged as a method for fast, quantitative Magnetic Resonance Imaging. This paper reviews the current status of MRF up to early 2020 and aims to highlight the advantages MRF can offer medical imaging professionals. By acquiring scan data as pseudorandom samples, MRF elicits a unique signal evolution, or ‘fingerprint’, from each tissue type. It matches ‘randomised’ free induction decay acquisitions against pre‐computed simulated tissue responses to generate a set of quantitative images of T_1_, T_2_ and proton density (PD) with co‐registered voxels, rather than as traditional relative T_1_‐ and T_2_‐weighted images. MRF numeric pixel values retain accuracy and reproducibility between 2% and 8%. MRF acquisition is robust to strong undersampling of k‐space. Scan sequences have been optimised to suppress sub‐sampling artefacts, while artificial intelligence and machine learning techniques have been employed to increase matching speed and precision. MRF promises improved patient comfort with reduced scan times and fewer image artefacts. Quantitative MRF data could be used to define population‐wide numeric biomarkers that classify normal versus diseased tissue. Certification of clinical centres for MRF scan repeatability would permit numeric comparison of sequential images for any individual patient and the pooling of multiple patient images across large, cross‐site imaging studies. MRF has to date shown promising results in early clinical trials, demonstrating reliable differentiation between malignant and benign prostate conditions, and normal and sclerotic hippocampal tissue. MRF is now undergoing small‐scale trials at several sites across the world; moving it closer to routine clinical application.

## Graphical abstract

Magnetic resonance fingerprinting (MRF) is an emerging fast, quantitative magnetic resonance imaging technique, which has been shown in small studies to reliably differentiate between diseased and normal tissue. Siemens, Philips and GE Healthcare have published work on various aspects of MRF, indicating wider industry interest in MRF.

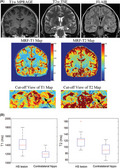

## Introduction

Magnetic Resonance Imaging (MRI) techniques exploit the response of protons in a strong external magnetic field (B_0_), to a radiofrequency (RF) pulse. The times taken for relaxation of proton spin precession in the longitudinal and transverse planes are called T_1_ and T_2_, respectively. These are tissue‐specific and can serve as biomarkers.

In current MRI techniques, the signal in each voxel is T_1_ or T_2_ weighted and shows a shade of grey reflecting its relative signal intensity, which can be described as ‘hyperintense’ or ‘hypointense’.^1^ Hardware and software differences can cause differing signal values in each voxel, thus current imaging techniques are largely qualitative. This poses a challenge for large‐scale longitudinal MRI studies, early detection and progress‐tracking of disease.^2^

Quantitative MRI aims to measure parameters like T_1_ and T_2_ in a reproducible manner and generate images with standardised contrasts. This will better reflect pathology at a cellular level,^3^ reduce subjectivity, enable direct comparison of images,^4^ and help radiologists characterise lesions and make more informed diagnoses.^5^ Current quantitative MRI methods require long acquisition times which are not clinically practical.^2^

**Figure 1:**
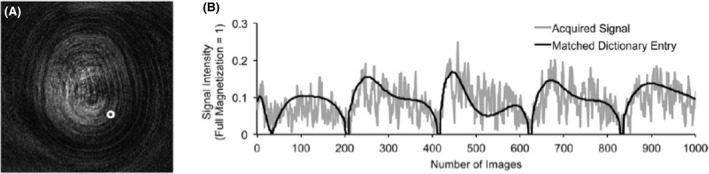
(A) An example of the undersampled images from MRF‐FISP. (B) A representative time course of one pixel, as indicated by the white circle in (A) and its matched dictionary entry. The estimated T_1_ and T_2_ values of this pixel are 750 ms and 65 ms, respectively. The longitudinal axis represents the fraction of the full magnetisation that is equal to one. FISP, fast imaging with steady‐state precession; MRF, magnetic resonance fingerprinting. Reproduced with permission from Jiang et al.^3^

Instead of preparing the system to obtain steady‐state signals as in conventional MRI, magnetic resonance fingerprinting (MRF) uses pseudorandom acquisition parameters for radiofrequency flip angles (FA) and repetition times (TR) to best elicit the full range of combined T_1_, T_2_ and proton density (PD) information obtained as the scan progresses. The transient‐state signals or ‘fingerprints’ are characteristic of tissue types. They are captured as undersampled images per time point,^6^ and matched with a dictionary,^3^, ^5^ which is akin to a large look‐up table of pre‐computed templates of signal responses (Fig. 1). The dictionary contains profiles of all likely resonance signals simulated using the Bloch equations or extended phase graph formalism.^3^, ^7^ This choice assigns absolute T_1_, T_2_ and PD values, facilitating quantitative tissue characterisation.

Undersampling k‐space for each time point enables scan acceleration without compromising image quality.^1^ MRF has some tolerance to undersampling and motion artefacts, which are not translated to the final image provided the samples are spatiotemporally incoherent. Other artefacts are also tolerated if they do not mimic a valid ‘fingerprint’ or cause ambiguous matching with dictionary parameters.^1^, ^5^

Beyond altering the MRI signal acquisition sequences, moving from current standard MRI protocols to adopt MRF imaging will require several procedural changes. MRF data from each patient must be matched to a dictionary, which needs to be computed rapidly, accurately and reliably. Accelerated image acquisition under MRF protocols means different types of image artefacts may arise. This needs to be monitored and suppressed by suitable techniques where possible.

Several review papers^5^, ^8^, ^9^, ^10^, ^11^ have been published on MRF in recent years, summing up the advances and refinement of the technique.

This paper considers the advances in MRF image acquisition, developments in image matching, reconstruction and artefact suppression, clinical applications and near‐term potential impact of MRF. We aim to bring medical imaging professionals up to date with this technique, which could see clinical implementation in the near future.

## Methodology

For this literature review, papers were obtained from Scopus database searches between July 2018 and January 2020. Inclusion criteria were: articles in English containing key words including ‘magnetic resonance fingerprinting’, ‘MR fingerprinting’ and ‘MRF’. Papers citing the original^1^ MRF article were also examined. While emphasis was placed on papers published in 2018 or later, earlier papers that described either important acceleration techniques or clinical applications were also included. Papers covering technical advances, such as sequence optimisation and dictionary search optimisation techniques, were excluded from this review, regardless of age.

**Figure 2:**
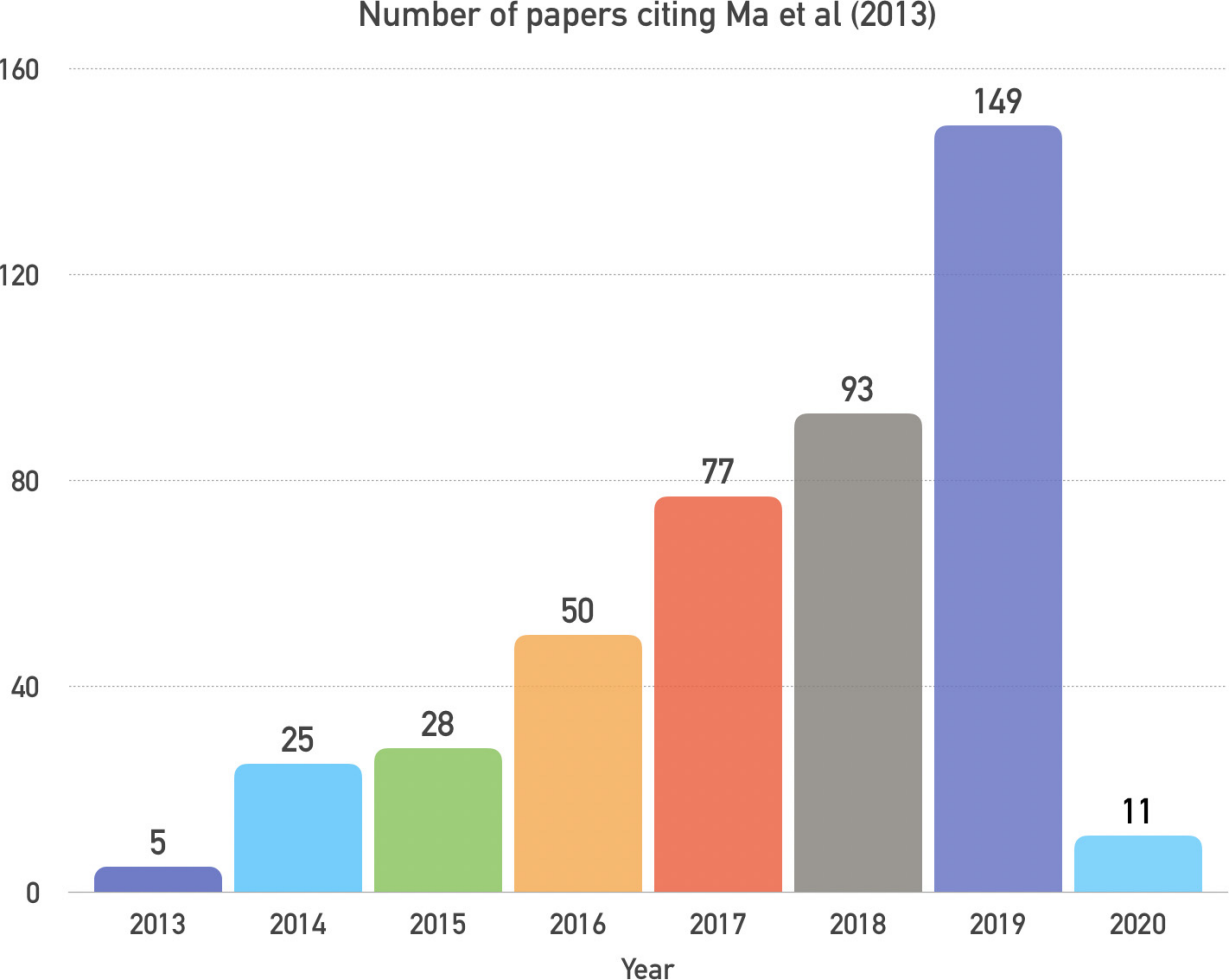
Number of papers referencing Ma et al.^1^ to 22 Jan 2020 organised by year, used as a proxy to measure interest in MRF. Statistics obtained from Scopus Metrics.^12^

MRF research has grown rapidly (Fig. 2). As of 22 January 2020, 438 papers have referenced the original paper, placing it in the 99th percentile for papers in Medicine over the same period.^12^ MRF research published since 2013 has consolidated and covered in depth a range of topics, including sequence optimisation, artefact reduction, accuracy and clinical applications.

## Acquisition Approaches

### Overview

**Figure 3:**
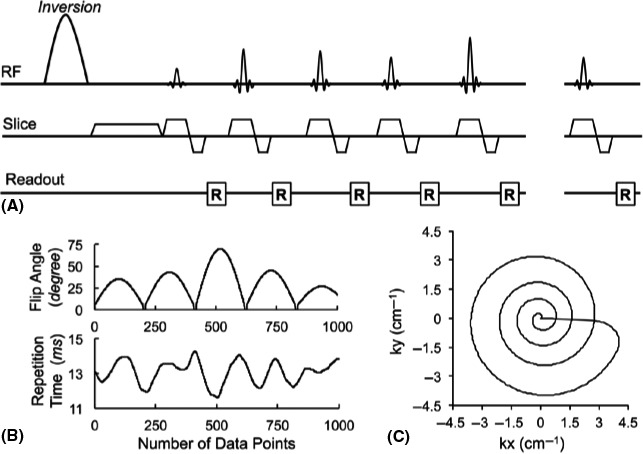
(A) A pulse sequence diagram of the MRF‐FISP sequence. An adiabatic inversion pulse is followed by a series of FISP acquisitions. (B) A sinusoidal variation of flip angles and repetition times in a Perlin noise pattern, are used in the MRF‐FISP sequence. (C) One interleaf of a variable density spiral is used in each repetition. The spiral trajectory is zero‐moment compensated. It needs 24 interleaves to fully sample the centre of the k‐space, and 48 interleaves for 256*256. The trajectory rotates 7.5 degrees every repetition. Reproduced with permission from Jiang et al.^3^

The original MRF sequence was based on balanced steady‐state free precession (bSSFP) but had banding artefacts caused by B_0_ inhomogeneities.^1^ Later studies overcame these artefacts using MRF‐Fast Imaging with Steady state Precession (FISP) with an unbalancing gradient moment after each TR to retain signal coherence (Fig. 3).^3^, ^5^ MRF‐FISP is fast and accurate, scanning a 256 x 256 slice in 13s and deviating less than 1% from gold standards for T_1_ and T_2_. It has since been used as a basis for several MRF sequences trialled for clinical use.^13^, ^14^, ^15^, ^16^, ^17^, ^18^, ^19^

### Overcoming RF field (B_1_^+^) inhomogeneity and implant‐associated issues

B_1_^+^ and B_0_ inhomogeneities are a common cause of artefacts and can cause inaccuracies in quantitative parameter estimation. B_1_^+^ and B_0_ inhomogeneities may be caused by inherent imperfections in MR equipment, and the latter in particular can be amplified by in vivo metallic implants.

B_1_^+^ inhomogeneity causes varying effective FA in the region of interest, resulting in inaccurate parameter quantification at higher field strengths.^20^, ^21^, ^22^ One measure to counter this used a FISP‐based MRF sequence with abrupt FA changes and included a B_1_^+^ dimension in the dictionary simulation to improve T_2_ accuracy.^7^

Plug‐and‐Play MRF used heterogeneous but complementary RF fields to eliminate B_1_^+^ voids, producing detailed property maps in the presence of a titanium orthopaedic implant.^23^ The B_1_^+^ distribution was co‐encoded into the MR fingerprints, enabling their spatial variations to be factored out in a single image reconstruction process.

In another approach, the Quick Echo Split Imaging technique was combined with MRF to scan with fewer, low amplitude RF pulses.^4^ It accurately quantified T_1_, T_2_ and PD with minimal RF power deposition, paving the way for the use of MRF in ultra‐high fields, or in patients with metallic implants.

### Undersampling, scan acceleration and volume acquisition

**Figure 4:**
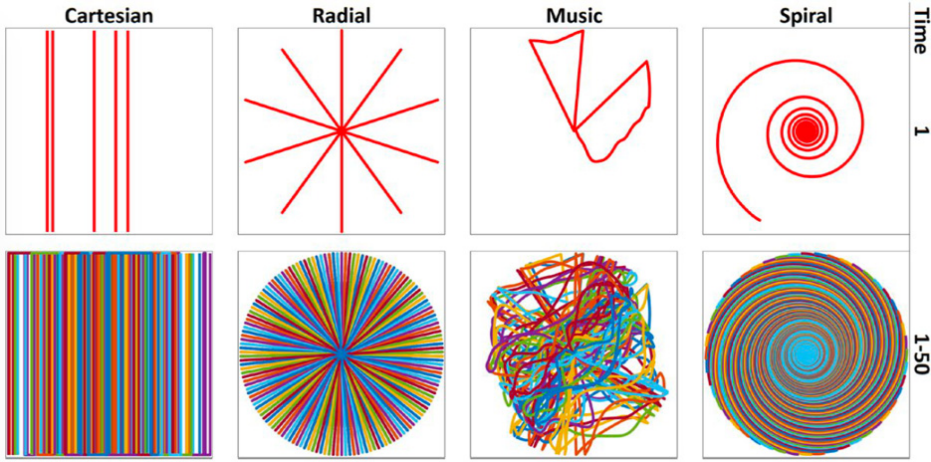
Examples of k‐space sampling trajectories used by different MRF sequences. (A) Cartesian. (B) Radial. (C) Trajectory generated from a music file (Yo Yo Ma playing Johann Sebastian Bach’s Cello Suite No. 1) for the MRF‐Music design. (D) Variable density spiral. For each time point, the trajectory changes to generate undersampling artefacts that are incoherent with the tissues’ fingerprints. Adapted with permission from Mehta et al.^5^

k‐space sampling trajectories tested in MRF include spiral,^1^, ^3^, ^4^, ^16^, ^17^ radial,^24^ echo‐planar imaging,^25^ Cartesian^7^, ^26^ and music^27^ (Fig. 4).

While many MRF acquisitions, including the original study,^1^ undersample k‐space at rates from 4 up to 144^15^ to accelerate scans, others like perfusion^28^ and vascular studies^29^ do not, for image stability and artefact reduction.^5^

3D FISP‐MRF^15^ added phase‐encoding lines along the k_z_ direction to 2D MRF for scan acceleration, with correction for B_1_^+^ inhomogeneities. Whole‐brain quantitative 3D maps with a resolution of 1.2 × 1.2 × 3 mm^3^ were obtained in 4.6 min, with validation of T_1_ and T_2_ against the International Society for Magnetic Resonance in Medicine/National Institute of Standards and Technology (ISMRM/NIST) phantom.

## Dictionary Generation and Pattern Matching

Most MRF studies use the dictionary method proposed by Ma et al.^1^ to translate scan data into images, but promising, rapid non‐dictionary methods are also being explored.

As image reconstruction consists of matching signal evolutions from each voxel with simulated tissue properties in the dictionary, its accuracy is dependent on the MRF signal model used for dictionary simulation. The dictionary stores values quantised into variable‐sized steps across each parameter and must cover a comprehensive range of relevant combinations of tissue properties.^5^ Since the addition of each dimension causes an exponential increase in dictionary size,^30^ the desire to account for as many properties as possible must be balanced against dictionary size. As pattern matching can be time‐consuming with large dictionaries, efforts have been made to streamline this through optimisation methods.

The original MRF publication^1^ used a vector‐dot product of the signal with each simulated fingerprint in a straightforward template matching process. The dictionary entry with the highest dot product was deemed the best match, and the parameters associated with that entry were assigned to the voxel.^8^ However, dot product‐based measures may be susceptible to artefact‐induced matching errors,^31^ so stable acquisition sequences should be used.

## Optimising Image Reconstruction

### Dictionary methods

Reducing reconstruction time is important for clinical implementation of MRF as current unoptimised methods may take several minutes per slice.

In their comprehensive technical review, Mehta et al.^5^ covered the many methods to optimise and accelerate the dictionary‐matching process, including single value decomposition, low‐rank approximation, fast group matching and compressed sensing.

### Non‐dictionary image reconstruction methods

Unlike dictionary‐based grid search, non‐dictionary reconstruction methods estimate a continuum of MR parameters and do not suffer from grid quantisation bias^32^ or the ‘curse of dimensionality’.^30^ Several protocols employ faster scan times, although computing requirements and reconstruction times vary. Approaches tested include using a Kalman filter,^33^ treating quantitative MRI as a nonlinear tomography problem,^30^ kernel ridge regression,^32^, ^34^ and Deep Learning.^11^, ^35^, ^36^, ^37^, ^38^, ^39^, ^40^, ^41^, ^42^, ^43^, ^44^

Deep Learning in particular has shown promise, reducing errors in relaxometry estimates,^39^, ^40^, ^42^ and optimising the dictionary‐matching process,^36^, ^41^, ^43^ In one study, a four‐layer neural network utilising rapid feed‐forward processing was trained on simulated MRI data and tested on numerical and ISMRM/NIST MRI phantoms. Image reconstruction was accurate and demonstrated image reconstruction up to 5000 times faster, vast storage savings and robustness to noise as compared to conventional MRF dictionary‐matching.^36^

Hamilton and Seiberlich^11^ have published an overview of current research that combines MRF and machine learning, and how machine learning can speed up dictionary generation for cardiac MRF. McGivney et al. have also covered Deep Learning in MRF in their review,^9^ indicating that the original MRF authors are aware of the potential of Deep Learning for future development of the technique.

The power of Deep Learning stems from the quality, size and breadth of the data sets used for training. Training will benefit from regular updates as the breadth of patient cases grows with the increasing uptake of clinical MRF.

## Artefact Reduction

Despite MRF’s overall robustness to artefacts, partial volume and motion artefacts can still occur.

### Partial volume artefacts

Partial volume artefacts, which also occur in other volumetric acquisition methods such as computed tomography and conventional MRI, can be diminished with multicompartment models.^2^, ^45^, ^46^ In particular, Nagtegaal et al.^46^ used compressed sensing optimisation and sparsity techniques to model voxels of multicompartment tissue without making restrictive assumptions. This resulted in a robustness to noise that enabled tighter classification bounds on compartment fractions.

### Motion artefacts

Motion artefact reduction would improve cost and throughput in the clinical implementation of MRF by limiting the need for sedation or repeat scans.^47^ In‐plane and through‐plane motion during different parts of the scan had varying effects on T_1_ and T_2_ maps.^6^, ^48^ While end‐scan movements had less effect on parameter maps, this caused more relaxometry data points to fall outside the 95% confidence interval.^6^

Methods used to mitigate rigid body motion artefacts include an iterative reconstruction‐based retrospective motion approach dubbed ‘MORF’^47^, and a combination of sliding window reconstruction, rigid body image registration, k‐space motion correction and low‐rank reconstruction in MC‐MRF^48^.

**Figure 5:**
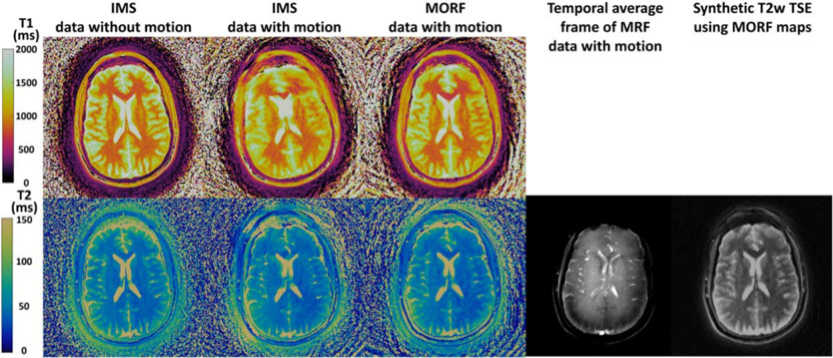
Reconstruction results from a prospectively motion corrupted in vivo experiment. For this experiment the subject was requested to move randomly according to his/her will. First column: IMS reconstruction maps from a separate scan without subject motion. Second column: IMS reconstruction maps using data with subject motion. Third column: MORF reconstruction maps using data with subject motion. Fourth column: Temporally averaged frame of the raw MRF data with subject motion. Fifth column: Synthetic T2w‐TSE image generated from maps reconstructed using MORF from data with motion. IMS results present a severe amount of motion artefacts not only in the T1 map but also in the T2 map. MORF results present significantly fewer artefacts compared with IMS and closely resemble the results from scan without motion. The subject ended up moving in the beginning ~ 17% of the acquisition as well as at the ending ~ 37% of the acquisition with a total of ~ 54% of data being motion corrupted. Adapted with permission from Mehta et al.^47^

MORF^47^ effectively removed in‐plane rigid body motion artefacts: images closely resembled non‐motion control images even when 54% of acquisition data was corrupted (Fig. 5). However, both MORF and MC‐MRF showed limited image correction with through‐plane motion artefacts^47^, ^48^.

Regularly Incremented Phase Encoding‐MRF (RIPE‐MRF)^26^ attempted to mitigate pulsatile and respiratory motion artefacts using altered k‐space trajectories to add temporal incoherence to motion artefacts, and used view‐ordering to minimise the impact of motion on quantitative maps. Compared to standard Cartesian MRF, RIPE‐MRF produced significantly reduced artefact‐to‐noise ratios, visibly fewer artefacts and improved uniformity. Despite bias in T_1_ and T_2_ estimates, RIPE‐MRF could eliminate the need for physiological gating and/or triggering to obtain artefact‐free quantitative maps.

## Clinical Applications

MRF has been trialled in brain,^14^, ^19^, ^45^, ^49^ abdomen,^16^ prostate,^50^, ^51^ vascular,^18^, ^28^, ^29^, ^52^, ^53^ cardiac,^17^, ^54^ musculoskeletal,^55^, ^56^ eye^57^ and breast^58^ applications. While initial results have shown that MRF‐generated quantitative relaxometry maps can differentiate between healthy and diseased tissues,^14^, ^16^, ^19^, ^45^, ^49^, ^50^, ^56^, ^57^, ^58^ most of these studies involved fewer than 100 subjects. Studies of larger cohorts of normal and pathological cases will be necessary for MRF to be validated for population‐relevant clinical implementation. Additionally, MRF can be performed in far less time than conventional MRI, which can reduce patient discomfort and increase scanner throughput. The speed of MRF demonstrated by the quantification of T_1_ and T_2_ values within a 19‐second breath hold^16^ make it highly applicable to abdominal imaging or even restless patients.

### Brain

Small‐scale MRF studies on the brain have been conducted on epilepsy,^14^, ^45^ tumours^19^, ^59^ and dementia^49^ and demonstrate the potential for tissue characterisation and tumour classification.

**Figure 6:**
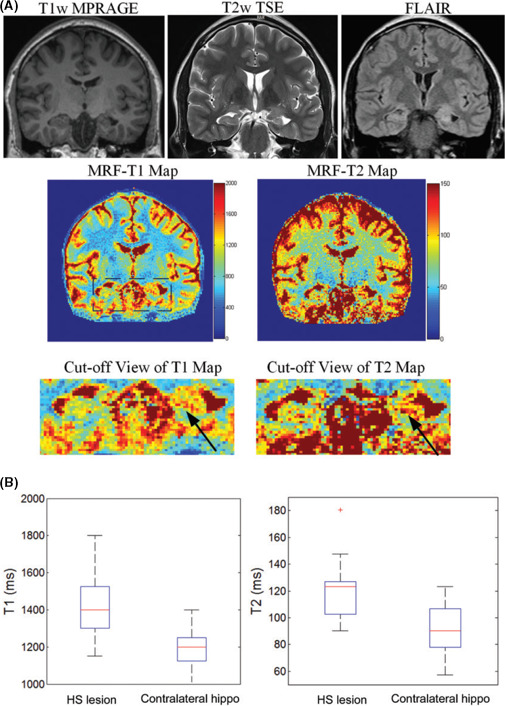
(A) Coronal position of T_1_‐weighted magnetisation‐prepared rapid gradient echo (MPRAGE), T_2_‐weighted fast spin echo (TSE), fluid‐attenuated inversion recovery (FLAIR), and T_1_ and T_2_ maps obtained by MR fingerprinting (MRF) in a typical patient with unilateral hippocampal sclerosis (HS; S14; 22‐year‐old man). Arrows on the T_1_ and T_2_ maps indicate the possible HS lesions. (B) Box‐and‐whisker plots of HS lesion and contralateral hippocampus (hippo). ms = msec. Reproduced with permission from Liao et al.^14^

A study comparing 2D MRF with conventional MRI epilepsy protocols found the former significantly faster, more accurate and more sensitive to subtle changes than conventional MRI (Fig. 6).^14^ Another epilepsy study^45^ used 3D FISP‐MRF to construct isotropic 1.2 mm^3^ voxels in 13.5 min, halving the scan time while also identifying subtle lesions not previously noted on conventional MRI.

Atrophied tissue in brain regions associated with dementia was found to have longer tissue relaxometry values than normal. The same study also found a possible correlation of the degree of deviation from normal relaxometry values with disease duration and severity.^49^

### Cardiac tissue

Acquisitions are performed during breath holds in cardiac MRF (cMRF). It also uses electrocardiogram‐triggering to minimise motion artefacts,^17^ introduces delays between acquisition periods in each heartbeat^54^ and uses extensive preparation pulses before each acquisition. Unlike MRF in other body parts, generation of subject‐specific dictionaries is required to account for heart rate variations.^17^

**Figure 7:**
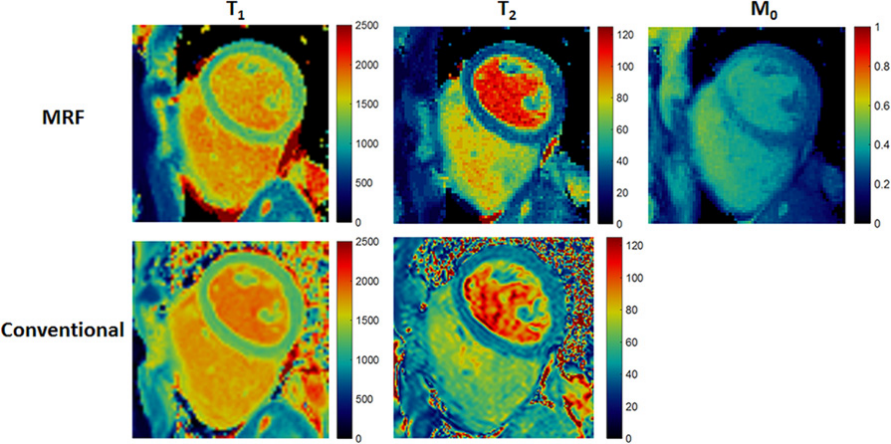
Maps from one volunteer of T_1_, T_2_ and M_0_ (PD) acquired with MRF (top row), MOLLI (bottom left) and a balanced SSFP sequence with three T_2_ preparation times (bottom middle). Reproduced with permission from Hamilton et al.^17^

A FISP‐based cMRF sequence was shown to acquire relatively accurate T_1_, T_2_ and PD maps in four heartbeats (Fig. 7).^17^ However, dictionary generation took 12s for each acquisition, and iterative reconstruction took almost 8 min per slice; this at present is not fast enough for clinical use. Nonetheless, initial clinical validation results are promising, with reliable differentiation between normal and diseased tissue, reduced artefacts and good reproducibility.^60^

A comprehensive overview of recent developments in cMRF can be found in Cruz et al.’s paper.^60^

## Potential Implementation and Future of MRF

### Repeatability and reproducibility in MRF

MRF parameter estimates must be repeatable and accurate, so that variations from the norm in that tissue can be confidently attributed to pathology.

**Figure 8:**
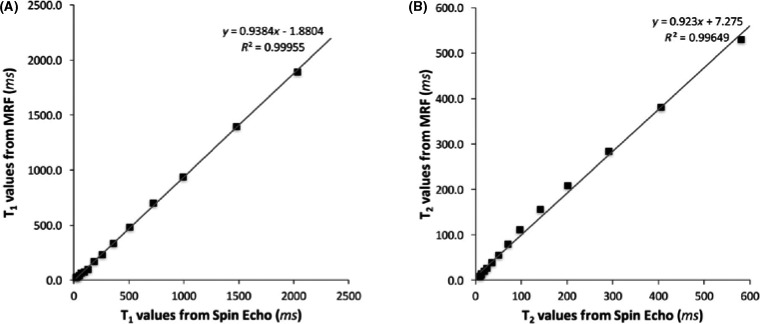
Correlation plots (A, B) comparing T_1_ and T_2_ values averaged over 34 consecutive days of MRF measurements to the T_1_ and T_2_ values obtained from the inversion recovery spin echo and multiple single‐echo spin echo methods, respectively. The results show a strong linear correlation (*R* ^2^ = 0.999 for T_1_, *R* ^2^ = 0.996 for T_2_). Reproduced with permission from Jiang et al.^61^

The ISMRM/NIST system phantom was imaged over 34 days using FISP‐MRF in one study.^61^ The relaxometry estimates were consistent with SE ones and demonstrated less than 5% variation throughout the experiment, except for short T_2_ times, which had less than 8% variation (Fig. 8). MRF parameter maps also had superior spatial resolution to SE.

In vivo studies have also indicated good reproducibility of MRF results. A study^15^ of six brain regions in five volunteers using 3D FISP‐MRF demonstrated the stability of T_1_ and T_2_ values obtained across subjects.

Separate in vivo MRF studies on hip cartilage,^55^ brain,^62^, ^63^ cardiac tissue^64^ and breast^65^ showed good repeatability and reproducibility across scanners. In particular, repeatability coefficients of variation for T_1_ and T_2_ in the brain study were below 8% and 14%, respectively,^62^ while it was better in breast tissue, ranging from 3 to 4% for T_1_ and 5 to 7% for T_2_.^65^ Another repeatability study in the brain^63^ found more variation of relaxometry values in cerebrospinal fluid compared to solid brain tissue, indicating that additional optimisation work may be needed for T_1_ and T_2_ quantification in pulsating fluids.

As all the reproducibility and repeatability studies involved relatively small samples, it would be essential to conduct further studies on larger groups, to generalise findings to the wider population. Additionally, there is still some way to go to achieve 1% consistency in parameter values, to make tissue relaxometry a reliable biomarker.

### Phantoms, standards and centre accreditation

To be a truly quantitative measure of tissue relaxometry properties, MRI quantitative values must be accurate and consistent between subjects, sites, scanners, protocols and over time.^66^ However, factors including hardware and software differences between scanners affect the accuracy of relaxometry measurements. Subject and tissue factors may also cause multi‐exponential or non‐exponential relaxation. Thus, pragmatic definitions are needed for relaxation times to be used as biomarkers for tissue type and pathology.^66^

Existing MRI phantoms like the American College of Radiology (ACR) phantom were designed for use with qualitative MRI and are not suited for use with MRF as their long‐term stability was not monitored.

Thus, Keenan et al.^66^ recommended a standard system phantom for quantitative MRI with SI‐traceable components, and verifiable long‐term accuracy and stability. Besides assessing system signal‐to‐noise‐ratio, resolution, geometric distortion and relaxation times, it would allow comparison of results across manufacturers, field strengths and hardware and software versions. To ensure scanners meet standards, an accreditation process, similar to the ACR MR Accreditation Program^67^ for qualitative MRI, may be needed for sites planning to offer MRF.^66^

In addition to repeatability and reproducibility studies, large‐scale in vivo studies will be needed to set benchmarks for parameter precision at different field strengths, standard time point lengths, k‐space undersampling and types of sequences suitable for clinical use.

### Professional acceptance of MRF and implications for radiographers

Full confidence in diagnoses based on quantitative relaxometry measurements will require the establishments of rigorous standards and a quality control framework to consolidate the clinical use of MRF. Large coordinated and standardised in vivo studies are needed to convince the imaging community that MRF quantitative maps can be used to make reliable diagnoses.

Radiologist input will be required for noise characterisation and presentation of relaxometry parameter maps. Implementing MRF would necessitate instructing radiographers on scanning protocols and optimisation methods, and training for radiologists to correlate deviations in tissue relaxometry with pathology.

Quantitative MRF will present new challenges for radiographers. Longitudinal or repeat examinations of a patient may require accurate co‐location of tissue voxels from previous MRF scans to permit quantitative tracking of time‐ or treatment‐evolving tissue response. Rigid and non‐rigid 3D image registration methods, like those used currently in fMRI, may become standard protocols.

Large‐scale implementation of MRF can only occur with sufficient buy‐in from hospitals and radiologic clinics. Stakeholders will need information on how it works, and its advantages and shortcomings. Pooling of expertise across hospitals and health systems, as well as standardisation of protocols, should also be encouraged. Early adoption of MRF would boost local clinical experience in the technique.

### Future of MRF

A recent paper by Assländer^68^ confirms our view that MRF researchers still need to overcome some practical and technical challenges before widespread implementation can occur. However, Siemens have provided many in the original MRF team with research grants,^16^ and have been involved in recent work.^15^, ^30^, ^40^, ^59^, ^63^ Philips^46^, ^48^, ^49^, ^69^ and GE Healthcare^41^, ^62^ researchers have published work relating to MRF, indicating wider industry interest.

Machine learning computation has advanced greatly in the last two years, opening avenues for researchers to propose faster and more precise capture and matching of MRF signals. Improved MRF methods to image smaller voxels using shorter scan times, and better model the response of voxels containing multiple tissue types, will undoubtedly generate great interest among medical imaging professionals.

In Australia, MRF is enabled on several scanners at the University of Queensland, the Herston Imaging Research Faculty, and the Commonwealth Scientific and Industrial Research Organisation, where we believe preliminary clinical trials are underway. To date, staff at the Florey Institute of Neuroscience and Mental Health in Victoria have contributed to several papers^45^, ^47^ on MRF research.

## Conclusion

MRF is a promising fast quantitative MRI technique that reliably differentiates between normal and diseased tissue. Besides expanding its utility to other body systems and pathology, protocols must be set for ensuring parameter precision is achieved at different field strengths so relaxometry values can be used as accurate biomarkers. Standards in pathology identification should be established through scanning large cohorts of healthy individuals and patients, to maximise the real benefits of clinical MRF. Finally, clinical centres offering MRF must be accredited to ensure the fidelity of quantitative results across sites.
